# Older *Thinopyrum intermedium* (Poaceae) plants exhibit superior photosynthetic tolerance to cold stress and greater increases in two photosynthetic enzymes under freezing stress compared with young plants

**DOI:** 10.1093/jxb/erw253

**Published:** 2016-07-08

**Authors:** Nikhil S. Jaikumar, Sieglinde S. Snapp, Thomas D. Sharkey

**Affiliations:** ^1^Institute for Genomic Biology, University of Illinois Urbana-Champaign, 1206 West Gregory Drive, Urbana, IL 61821, USA; ^2^Program in Ecology, Evolutionary Biology and Behavior, Michigan State University, East Lansing, MI 48824, USA; ^3^Department of Plant, Soil, and Microbial Science, Michigan State University, 1066 Bogue Street, East Lansing, MI 48824, USA; ^4^Department of Biochemistry and Molecular Biology, Michigan State University, 603 Wilson Road, East Lansing, MI 48824, USA

**Keywords:** Age, chilling, cold, freezing, perennial, photosynthesis, *Thinopyrum*.

## Abstract

Effects of plant age on resource acquisition and stress tolerance processes is a largely unstudied subject in herbaceous perennials. In a field experiment, we compared rates of photosynthesis (*A*), ribulose-1,5-bisphosphate (RuBP) carboxylation capacity (*V*_Cmax_), maximum electron transport rate (*J*_max_), and triose phosphate utilization (TPU), as well as concentrations of Rubisco and sucrose-phosphate synthase (SPS) in 5-year-old and 2-year-old intermediate wheatgrass (*Thinopyrum intermedium*) under both optimal growing conditions and cold stress in early spring and autumn. This species is a relative of wheat undergoing domestication. An additional experiment compared photosynthetic rates in different cohorts at mid-season and under colder conditions. We hypothesized that photosynthetic capacity in older plants would be lower under favorable conditions but higher under cold stress. Our hypothesis was generally supported. Under cold stress, 5-year-old plants exhibited higher *A*, TPU, and temperature-adjusted *V*_Cmax_ than younger plants, as well as 50% more SPS and 37% more Rubisco. In contrast, at mid-season, photosynthetic capacities in older plants were lower than in younger plants in one experiment, and similar in the other, independent of differences in water status. Both cohorts increased *A*, temperature-adjusted TPU and *J*_max_, [Rubisco], and [SPS] under cold stress, but changes were greater in older plants. Photosynthetic differences were largest at 1.2 ºC in very early spring, where older plants had 200% higher *A* and maintained up to 17% of their peak photosynthetic capacity. We find evidence of increased cold tolerance in older cohorts of wheatgrass, consistent with a growing body of research in woody perennials.

## Highlight

We explore interactive effects of plant age and cold stress on photosynthetic rates and key photosynthetic enzymes in a herbaceous perennial in the field.

## Introduction

A major knowledge gap in the ecological literature concerns how plant age affects resource acquisition processes in herbaceous perennials. Effects of whole-plant age on photosynthetic rate, as distinct from leaf age, have been studied in many perennial trees and shrubs. Conifers generally show a decrease in photosynthetic rate when seedlings are compared with young trees (e.g. Grulke and Miller, 1994), as do some deciduous species (Fredricksen *et al.*, 1996; Steppe *et al.*, 2011); other deciduous trees including aspen and beech show increases with age (Herbinger *et al.*, 2005; Pärnik *et al.*, 2014). One set of explanations offered for age-related changes in photosynthetic traits involves changes in optimal evolutionary strategy (Bond, 2000). Because small seedlings experience higher mortality risks than older plants, an optimal strategy for very young plants might favor photosynthesizing as rapidly as possible to escape the critical seedling stage. Conversely, older plants with a lower overall risk of death may prioritize stress tolerance over growth, and sacrifice photosynthetic capacity for greater tolerance against stresses such as cold, drought, and herbivory. In support of this idea, some evidence suggests that older individuals may show superior cold tolerance to juvenile and younger mature plants. Killing temperatures in *Rhododendron* decrease by 6–8 ºC in a single year of aging (Lim *et al.*, 2014). Likewise, age is linked to lower killing temperatures in *Phellodendron* (MacNamara and Pellet, 2000), and less cold-induced winter photoinhibition in mistletoe (*Viscum* spp.; Miguez *et al.*, 2014). Trade-offs exist between freezing tolerance and capacity for biomass accumulation (Gusta and Wisniewski, 2013; Wingler, 2014), which could lead individuals of certain species to invest more in freezing tolerance and less in growth or reproduction as they age.

In contrast to woody perennials, few studies have considered whole-plant age effects on photosynthetic traits in herbaceous perennials. Exceptions include nettle (*Urtica dioica*; Oñate and Munné-Bosch, 2009), giant *Miscanthus* (Boersma *et al.*, 2015), and yellow cryptantha (*Cryptantha flava*; Casper *et al.*, 2006; Salguero-Gomez and Casper, 2011), which show conflicting, species-specific trends. Above-ground tissues of herbaceous perennials, unlike woody species, largely re-grow anew each season. However, it cannot be assumed that they will show no age-related physiological effects. Above-ground tissues are linked to below-ground organs which survive from year to year, and may experience differences in nutrient availability, carbon budgets, water relations, sink strength, trade-offs between competing stresses, or pathogenic buildup as the plant ages. Therefore, characterizing changes in photosynthetic traits with whole-plant age, as well as age-related effects on stress tolerance, remain important challenges for herbaceous perennial species.

This study examines effects of plant age on photosynthetic traits in intermediate wheatgrass (*Thinopyrum intermedium*, syn. *Agropyron intermedium*), comparing photosynthetic traits both under favorable conditions at mid-season as well as under low temperature stress (a period of freeze–thaw cycles) early and late in the season. We considered newly emerged leaves on plants of varying ages, so as clearly to separate effects of leaf age from plant age. Intermediate wheatgrass is a Eurasian perennial C_3_ grass, closely related to wheat (*Triticum aestivum* and *Triticum durum*), currently naturalized across North America, with high photosynthetic rate (Jaikumar *et al.*, 2013) and biomass productivity (Culman *et al.*, 2013), and a lifespan of up to 50 years. *Thinopyrum* spp. have been extensively used in the past as breeding sources of disease resistance, photosynthetic improvement, and stress tolerance traits for wheat: this particular species is widely grown for forage and is currently under selection for higher yields of its edible, high-protein seeds, as a perennial food crop (Cox *et al.*, 2010; DeHaan *et al.*, 2014). As a highly cold-hardy species which maintains green leaves through the winter (Limin and Fowler, 1988), it also provides the opportunity to consider how plant age might interact with chilling and freezing stress in affecting photosynthetic traits. The high photosynthetic capacity, cold tolerance, and economic importance of this species make it a well-suited herbaceous perennial model to study age-related changes in photosynthetic traits, and their interaction with abiotic stresses.

Our guiding hypothesis was that as wheatgrass plants age, their optimal resource use strategy will shift from emphasizing the capacity for rapid growth under optimal conditions in favor of improving cold tolerance (Bond, 2000; Lim *et al.*, 2014). Wheatgrass is known to suffer yield declines over the first 5 years of growth, which is not ameliorated by nutrient addition (Canode, 1965), and this could represent a shift from a competitive to a stress tolerator strategy (Grime, 1977). If this is true, older plants might show lower photosynthetic capacity under optimal conditions and higher photosynthetic capacity under cold stress than younger plants. Because lower photosynthesis under warm conditions could also reflect a more conservative water use strategy rather than lower biochemical photosynthetic capacity, in one experiment we sampled leaf water potential (Ψ_L_), intrinsic water-use efficiency (IWUE), and stomatal limitation (*L*_S_) at mid-season to rule out this possibility. These changes in photosynthetic capacity should be reflected in changes in rates of photosynthesis under ambient conditions (*A*), maximum rate of ribulose-1,5-bisphosphate (RuBP) carboxylation (*V*_Cmax_), maximum electron transport rate (*J*_max_), and triose phosphate utilization (TPU) capacity, coupled with changes in concentrations of key enzymes including Rubisco (which limits *V*_Cmax_) and sucrose-phosphate synthase (SPS) which among other enzymes contributes to TPU capacity in Arabidopsis (Sun *et al.*, 2011), tomato (Galtier *et al.*, 1995), and tobacco (Rubio-Wilhelmi *et al.*, 2014). In wheat, increases in both enzymes are critical in cold acclimation (Savitch *et al.*, 1997). Our specific predictions were as follows. (i) Under favorable growing conditions at mid-season, older plants will show lower *A* as well as lower temperature-adjusted *J*_max_, *V*_Cmax_, and TPU than younger plants, but no increase in *L*_S_ or Ψ_L_ and no decrease in IWUE. (ii) During periods of freeze–thaw cycles in early spring and late autumn, older plants will show higher *A* and higher temperature-adjusted *J*_max_, *V*_Cmax_, and TPU than younger plants, indicating superior cold tolerance. (iii) Concentrations of Rubisco and SPS will follow the same trend: older plants will have higher levels at cold temperatures and lower levels under favorable growing conditions.

## Materials and methods

### Experimental goals and materials

We conducted two related experiments within a multi-year field trial, each comparing multiple wheatgrass age cohorts under cold and warm conditions. Experiment 1, carried out in 2013, compared *A* (assimilation under ambient conditions) in 1-, 2-, and 3-year-old plants under both intermittent freezing stress (April) and warmer conditions (May–July), as well as measuring leaf water potential Ψ_L_ under warm conditions. Experiment 2, carried out in 2015–2016, compared photosynthetic traits in two differently aged cohorts (2 and 5 years old at the start of the experiment) under both intermittent freezing stress (April 2015, November 2015, and March–April 2016) and optimal growing conditions (May–July 2015). We inferred *A*, *V*_Cmax_, *J*_max_, (*L*_S_), and TPU from *A*/*C*_i_ (photosynthetic carbon response) curves, and standardized values to 25 ºC (so as to separate changes in allocation under cold stress from temperature-linked changes in activity due to kinetic properties of the relevant enzymes). In this experiment, Rubisco and SPS levels were also compared in April and May.

The intermediate wheatgrass population we used (*Thinopyrum intermedium* ‘TLI-C1’) was a breeding population that had undergone one cycle of selection for grain yield and seed size at The Land Institute (Salina, KS, USA) and has been used for previous studies at this site (Culman *et al.*, 2013; Jaikumar *et al.*, 2013).

### Site

Our study was conducted at the W.K. Kellogg Biological Station of Michigan State University. The site is located in southwest Michigan, USA, 50 km east of Lake Michigan (42°24'N, 85°24'W, elevation 288 m), within the oak–maple–hickory forest/oak savanna transition zone. Soils are fine to coarse loamy, mixed mesic Typic Hapludalfs, developed from glacial outwash. The area receives 900mm of precipitation annually, approximately half as snow. Cumulative precipitation from 1 March to 1 June was 355, 217, 318, 200, and 200mm in 2011–2015, respectively, and 230mm for the 28 year average. Mean daily temperature over the period was 7.9, 12.0, 7.5, 6.9, and 8.6 ºC in 2011–2015, respectively, and 8.2 ºC for the 28 year average. Over the period of sampling in early spring 2016 (1 March– 10 April), the mean temperature was 4.0 ºC and precipitation was 152mm, compared with 2.9 ºC and 84mm for the 27 year average. As support for our selection of sampling dates, the frost-free period lasted from 26 April to 23 October in 2013, from 17 May to 10 October in 2014, and from 28 April to 17 October in 2015, and began on 13 April in 2016. Daily minima below 0 ºC typically continued through the last week of April (70% of the years between 1990 and 2016.)

### Experimental design and management

Our study formed a completely randomized design (CRD). Wheatgrass was planted in October of 2010, 2011, 2012, 2013, 2014, and 2015 at 175 seeds m^−2^, 2.5cm deep, in 2.30 m^2^ plots, with *n*=6 or *n*=4 depending on the year. Following each harvest, existing plots re-grew into the next season. Thus, in 2015, for example, the field included 1-, 2-, 3-, 4-, and 5-year-old wheatgrass, of which 2-year-old (*n*=4) and 5-year-old (*n*=6) plots (i.e. plants that are ~16 and 52 months old, respectively, in early March) were selected for the study. These plots were sampled again in early spring 2016, at which point they are referred to as 3- and 6-year-old plots, respectively. The field was fertilized each October with 91kg ha^−1^ N, 68kg ha^−1^ P_2_O_5_, 45kg ha^−1^ K_2_O, and 159 ha^−1^ Ca in the form of pelletized dried poultry manure (Herbruck Ranch, Saranac, MI, USA). In October 2014, these rates were increased 50% because of lack of mechanical incorporation. Plots were hand weeded as necessary during each season. Due to unusually hot and dry weather in July–August 2012, plants were irrigated with 180mm of water.

### General procedure for photosynthetic measurements

In both experiments, the same general procedure was used both to measure photosynthetic rate at ambient [CO_2_] (*A*) and to generate curves of photosynthetic response to intercellular [CO_2_] (*A*/*C*_i_ curves). In all cases, species and age classes were sampled in temporal blocks to cancel out temperature fluctuation. We used newly emerged, fully expanded leaves of 1–3 visibly healthy, deep green-colored plants per plot, using a LI-6400 XT portable gas-exchange system (LI-COR Instruments, Lincoln, NE, USA) with a gas-exchange head in 2013 and a combined fluorescence/gas-exchange head in 2015–2016. To avoid issues of changing source versus sink balance, only leaves on vegetative tillers were chosen, even at the summer dates. As weather permitted, we took the measurements during either the morning (08:30–12:30h) or the afternoon (14:00–16:00h), matching peak times for wheat photosynthesis (Rai *et al.*, 2011), setting photosynthetically active irradiance equal to 1200 µmol m^−2^ s^−1^, equivalent to mid-morning full sunlight in May. Chamber relative humidity was 55–75% and leaf temperatures were ~2 ºC above ambient: leaves were allowed to equilibrate until fluctuations in *A* were within 5%. In 2015–2016, fluorescence was measured with the modulated chlorophyll fluorometer. The multiphase flash fluorescence (MPF) protocol (Loriaux *et al*., 2013) was used to estimate maximum chlorophyll fluorescence in the light (*F*_m_') from which PSII efficiency as well as the ratio of variable to maximal fluorescence in the light (*F*_v_^'^ /*F*_m_^'^) was calculated. The 900ms long MPF intensity was 8000 μmol m^−2^ s^−1^ with a 20% phase 2 ramp.

### Photosynthetic traits under varying temperature conditions

In both experiments we measured photosynthetic traits during a range of temperature conditions, to determine how the performance of younger and older cohorts was affected by temperature. In 2013 (Experiment 1) we measured *A* on 13 April, 22 April, 18 May, and 9 July with leaf temperature ~8, 16, 23, and 30 ºC on these dates. We also generated light–response curves on May 18 to estimate light respiration (*R*_D_, data not shown) and generated *A*/*C*_i_ curves for first- and third-year plants on 16–18 May. In 2015 (Experiment 2) we generated *A*/*C*_i_ curves for fifth-year plants on 18 March, 24 March, 4 April, 8 April 26 April, 12 May, 10 July, and 20 November, and for second-year plants as well on the last five dates. Sampling in March was on overwintered leaves, and on the other dates was done on newly emerged leaves. Except on May and July dates, plants were entirely in vegetative stages: early vegetative until early April and late vegetative until early May. Previous observations demonstrated that after the first year, there were no discernible phenological difference between cohorts, thus 5- and 2-year-old plants were on the same phenological schedule (data not shown). Leaf temperature measured by the Licor thermocouple was ~4.9, 7.5, 2.8, 12.7, 6.8, 19.9, 28.6, and 7.5 ºC during measurement on these dates: all except the May–July dates were taken under conditions of cold stress (i.e. within the period usually characterized by freezing risk). With the exception of the July dates, all plants were in the vegetative stage.

As an additional part of Experiment 2, in early spring of 2016 we measured photosynthetic rate, fluorescence parameters, and stomatal conductance at three time points in very early spring: 14 March, 17 March, and 3 April. Three plants were sampled per plot as described above. The cohorts sampled in this experiment were the same as those sampled in 2015, but will be referred to (when discussing 2016 measurements) as ‘3-year-old’ and ‘6-year-old’ respectively. On these dates, leaf temperature measured with the Licor thermocouple was ~1.2, 9.7, and 4.7 ºC. This was of particular interest since the lowest temperature measurements in 2015 (at 2.8 ºC) had not included the younger cohort. While time did not permit determinations of *A*/*C*_i_ curves, we also measured photosynthetic rates at *C*_a_=2000 μmol mol^−1^ on 14 March, on the assumption (based on 2015 data) that at such low temperature, carbon-saturated *A* should be limited by and thus reflective of TPU.

### Photosynthetic carbon dioxide–response curves

To generate the carbon dioxide–response curves, we measured photosynthesis at *C*_a_=50, 75, 100, 150, 200, 250, 300, 400, 500, 600, 800, 1200, 1600, and 2000 μmol mol^−1^ CO_2_. *A* as a function of *C*_i_ was modeled as the minimum of RuBP carboxylation (Equation 1), electron transport (Equation 2), and TPU (Equation 3), using the method outlined in Long and Bernacchi (200) (see also Farquhar *et al.*, 1980; Sharkey *et al.*, 2007). Electron transport was converted to maximal electron transport capacity (*J*_max_) using Equation 4 based on light–response curves measured in May 2013. Chloroplast carbon dioxide concentration *C*_C_ can be related to *C*_i_ by Equation 5, and was used to estimate mesophyll conductance (*g*_m_: Equation 6) based on May 2015 measurements, with the assumption that *J*_F_=[incident light] [0.87] [0.5].

$$A=V_{\text{Cmax}}[C_{\text{C}}–\Gamma *]/[C_{\text{C}}+K_{\text{C}}(\text{1}+\left[{\text{O}}_{2}\right]/K_{\text{O}})]–R_{\text{D}}$$ (1)

$$A=J[C_{\text{C}}–\Gamma *]/[\text{4}C_{\text{C}}+\text{8 }\Gamma *]–R_{\text{D}}$$ (2)

$$A=\text{3TPU}+V_{\text{Cmax}}\Gamma */C_{\text{C}}–R_{\text{D}}$$ (3)

$$J=(J_{\text{max}}\alpha I–{\left[{\left(J_{\text{max}}+\alpha I\right)}^{\text{2}}–\text{4}J_{\text{max}}\alpha I\theta \right]}^{0.\text{5}})/(\text{2}\theta )$$ (4)

$$C_{\text{C}}=\Gamma *\;[J_{\text{F}}+\text{8 }(A+R_{\text{D}})]/[J_{\text{F}}–\text{4 }(A+R_{\text{D}})]$$ (5)

$$g_{\text{m}}=A/[C_{\text{C}}–C_{\text{i}}]$$ (6)

We assumed values of 34ppm at 25 ºC for the photocompensation point (Γ*) based on the related species western wheatgrass (*Pascopyrum smithii;* Dong *et al.*, 2012), and values of 272ppm and 166 000ppm for *K*_C_ and *K*_O_, respectively. Day respiration (*R*_D_) was fixed at 3.32mol m^−2^ s^−1^ at 25 ºC based on light–response curves taken in May 2013 (data not shown), and adjusted for temperature. The light–response curves suggested that the light level used (while equivalent to ambient light at this time of year and latitude) was slightly non-saturating; thus, we converted observed *J* to *J*_max_ values (at saturating light) based on the light–response curves (Equation 4). (The ratio of *J* to *J*_max_ did not differ between older and younger plants; therefore, we used the same conversion factor.) Mesophyll conductance was estimated at 8.1 μmol m^−2^ s^−1^ Pa^−1^ based on May 2015 data, and this value (following temperature correction) was used in fitting *A*/*C*_i_ curves. Temperature corrections for all parameters were based on Arrhenius or modified Arrhenius functions taken from Sharkey *et al.* (2007). Adjusting for temperature allowed us to compare the activity of components of the photosynthetic apparatus at various points in the growing season irrespective of actual temperature at the time.

Estimation of TPU capacity poses a common problem. Under many situations *in vivo*, TPU limitation is absent or difficult to observe. We observed TPU limitation in 82% of the plants (indicated by a decline in either *A* or Φ PSII at high *C*_i_), raising the question of how to handle missing data. These missing TPU values may be ‘imputed’ as the maximal photosynthetic rate achieved on the *A/C*_i_ curve (i.e. as their lower bound), as recent arguments have been made that plants typically maintain TPU capacity in only slight excess of electron transport capacity (Yang *et al.*, 2016). This may reduce the reliability of tests of statistical significance, however. Alternatively, plants with ‘missing’ values may be omitted and calculations done only on the plants where TPU was actually measured. This will downwardly bias estimated means, as well as potentially underestimating the degree of temperature-related acclimation by ignoring values that were too high to detect. We therefore chose a combined approach. Means in Table 3, overall *F*-tests, and estimates of the change in TPU under cold conditions are based on imputed values to give the reader a better sense of actual TPU rates in this species. To maintain appropriate statistical conservatism, though, comparisons between older and younger plants in cold and warm conditions are reported both with and without the imputed data.

### Rubisco and sucrose phosphate synthase

In 2015 (Experiment 2) we collected leaf samples on 8 April, 26 April, and 12 May, to assay concentrations of Rubisco and SPS. The first two dates were within the cold stress period (i.e. the period when late frosts are common); the third was not. Approximately 6cm^2^ of leaf tissue was clipped from newly emerged, fully expanded, visibly healthy leaves, halfway along the leaf. Leaf tissue from 3–4 plants per plot was composited, stored over dry ice and then at –80 ºC, ground under liquid nitrogen, and extracted in 1ml of phosphate-buffered saline (PBS). Rubisco was estimated via a competitive inhibition ELISA (Horsenall, 1984; Metodiev and Demirevska-Kepova, 1992; Orellana and Hansell, 2012), using an ELISA kit (LifeSpan BioSciences, Seattle, WA, USA). Rubisco in the sample competed with pre-coated Rubisco for anti-Rubisco binding, after which sample wells were washed and incubated with a secondary antibody conjugated to horseradish peroxidase. Sample wells were then re-washed, incubated with tetramethylbenzidine solution, and absorbance measured at 450nm and compared with a standard curve. SPS was assayed with a positive ELISA kit (MyBioSource, San Diego, CA, USA; www.mybiosource.com), following extraction in PBS, and absorbance at 450nm was compared with a standard curve (Walker and Huber, 1989; Chan *et al.*, 1990).

### Water relations

In Experiment 1, we quantified some basic water relations traits to verify whether any changes in observed photosynthetic rates were due to changes in water status rather than biochemical processes. Intrinsic water efficiency was calculated from IWUE=*A*/*g*_*S*_, with stomatal conductance (*g*_s_) measured simultaneously with photosynthesis. Stomatal limitation was calculated from *A*/*C*_i_ curves:

$$L_{\text{S}}=\text{1}–A_{\text{o}}/A_{\text{i}}$$ (7)

where *A*_o_ and *A*_i_ correspond to *A* at *C*_a_=400 and at *C*_i_=400, respectively, determined through linear interpolation (Farquhar and Sharkey, 1982). Water potential measurements were done on 12 July 2013 as we expected the warm weather to accentuate differences in water status. We selected flag leaves (at 16:00–16:30h) from healthy plants and used a pressure chamber (PMS Instruments, Corvallis, OR, USA) to measure pre-dawn and mid-day water potential (Ψ_L_).

### Statistical analysis

In Experiment 1, traits were analyzed separately at each date by a mixed-model ANOVA, with age as a fixed factor and ‘mature cohort age’ (i.e. differentiating between 2- and 3-year-old plants) as a nested fixed factor. For *V*_Cmax_ and *J*_max_, we compared the oldest and youngest cohort using an unpaired unequal-variance *t*-test. TPU was compared using a paired *t*-test due to large variation between blocks (*n*=6).

In Experiment 2 (assessing photosynthetic traits under cold stress), all data were analyzed using a CRD ANOVA with stress, date (nested within treatment), plant age, and the interaction of treatment and plant age as fixed factors. Here ‘stress’ represents the presence or absence of cold stress (i.e. the November and April samplings versus those in May–July). If there was a significant interaction term, we compared 5-year-old and 2-year-old cohorts separately under cold-stressed and unstressed treatments, combining all dates within each treatment. A planned contrast was also calculated, comparing the change in each parameter under cold stress in younger and older cohorts, for example ΔSPS=([SPS]_Cold_–[SPS]_Warm_])/[SPS]_Warm._ Photosynthetic measurements in early spring 2016 were considered separately: here we treated age, temperature, and the interaction of the two as fixed factors. At each temperature, the cohorts were compared. All comparisons between cohorts were done using Welch’s *t*-test following the Šidak–Bonferroni correction:

$$\alpha_{\text{adjusted}}=\text{1}–{\left[\text{1}-\alpha \right]}^{\text{1}/m}$$

for *m*=3 comparisons. Reported *P*-values are not adjusted, but we report only those that were significant at *P*=0.0169.

As described above, TPU was a special case because some values were too high to detect. Here we compared TPU (omitting any values too high to detect) between 2- and 5-year-old plants separately under warm and cold conditions: we ignored blocks, pooled all cold and all warm dates together, and performed an unpaired unequal-variance *t*-test following the Bonferroni correction for *m*=3 (df=23 under cold and df=17 under warm conditions). *F*-tests for significance were done using PROC MIXED in SAS 9.2 (SAS Institute, Cary, NC, USA). [Rubisco] was cube-root transformed, [SPS] was square-root transformed, and ΦPSII was logit-transformed to meet assumptions of ANOVA.

## Results

### Photosynthetic rates under cold stress and warm conditions (Experiment 1)

**Fig. 1.**
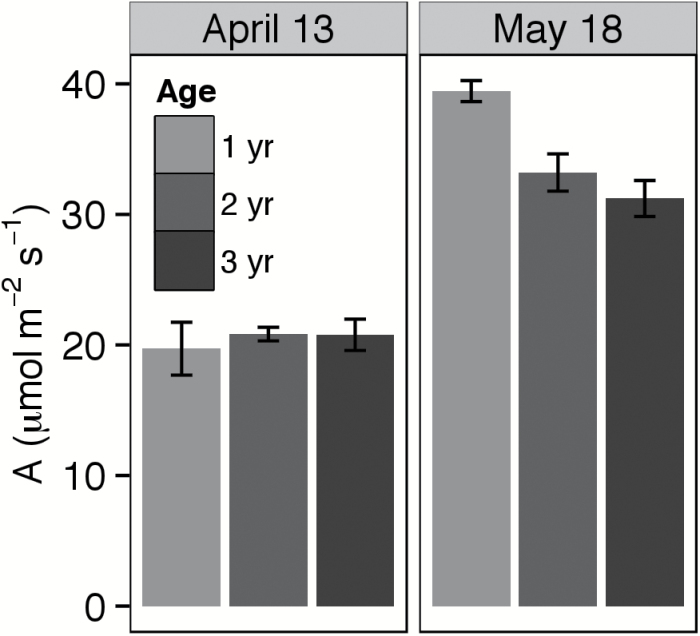
Observed photosynthetic rate at approximately ambient irradiance, [CO_2_], and humidity (*A*_o_) in 1-, 2-, and 3-year-old intermediate wheatgrass plants sampled at air temperature 8 ºC (on 13 April) and at air temperature 23 ºC (on 18 May). Data were taken from a 2013 study at the Kellogg Biological Station (Hickory Corners, MI).

In 2013, the age cohorts showed different photosynthetic trends at the four sampling dates. In early spring, age had no effect on photosynthesis. For example, *A* was ~18–23 μmol m^−2^ s^−1^ across all age cohorts in mid-April 2013, at which point first-year plants were in the early and older plants were in the early to middle vegetative stage. By mid-May, however, age-related differences became evident. On 18 May, *A* was ~37–42 μmol m^−2^ s^−1^ for first-year plants, and 18% lower for the older cohorts (*F=*9.71, *P*<0.01; Fig. 1). *A*_o_ did not differ between second- and third-year plants. Similarly, younger plants had higher photosynthetic activity in July but not in mid-April and July (Table 1).

**Table 1.** Means (±SE) and *F*-values for observed photosynthetic rate under ambient light and [CO_2_] (*A*), water-use efficiency (WUE), and for leaf water potential (Ψ_L_) at pre-dawn (PD) and mid-day (MD), measured on 1-, 2-, and 3-year-old cohorts of intermediate wheatgrass (Thinopyrum intermedium) in a 2013 study at Kellogg Biological Station (Hickory Corners, MI) Means for the two older cohorts are compared against first-year plants (*n*=6 for first- and third-year cohorts, *n*=4 for second-year).

	***A* (μmol m** ^**−2**^ **s** ^**−1**^)	**IWUE** (mmol mol^−1^)	***A* (μmol m** ^**−2**^ **s** ^**−1**^)	**IWUE** (mmol mol^−1^)	Ψ_L,PD_ (MPa)	Ψ_L,MD_ (MPa)
Date	22 April	22 April	12 July	12 July	12 July	July 12
Wheatgrass (1-year-old)	26.6±4.5	139.9±19.2	37.2±3.4	42.9±5.0	–0.16±0.01	-0.46±0.06
Wheatgrass (2-year-old)	26.7±0.9	55.6±8.2	29.3±2.2	43.1±2.7	–0.24±0.03*	-0.66±0.14
Wheatgrass (3-year-old)	24.2±1.6	72.3±3.7	30.7±2.2*	43.5±7.5	–0.17±0.02	-0.81±0.19
**Source of variation**						
Age	0.04	88.93***	21.06**	2.90	2.90	0.00
Mature cohort age	0.62	7.92*	2.01	0.04	6.35	0.94

*, **, and *** indicate significance at α=0.05, 0.01, and 0.001 respectively.

### Photosynthetic traits at mid-season (Experiment 1)

At mid-season 2013, under warm growing conditions, all three biochemical rates affecting photosynthesis showed clear declines with age. *V*_Cmax_ was 134–218 µmol m^−2^ s^−1^ in the youngest cohort and 98–157 µmol m^−2^ s^−1^ in the oldest (*t*=2.43, *P*=0.019, df=9), while TPU was 20–40 µmol m^−2^ s^−1^ in the youngest cohort and 16–29 µmol m^−2^ s^−1^ in the oldest (*t=*4.12, *P*=0.0046). Electron transport capacity was also lower in third-year plants (178–307 µmol m^−2^ s^−1^ compared with 216–425 µmol m^−2^ s^−1^: *t*=3.59, *P*=0.016).

### Biochemical determinants of photosynthesis under cold stress and warm conditions (Experiment 2)

**Fig. 2.**
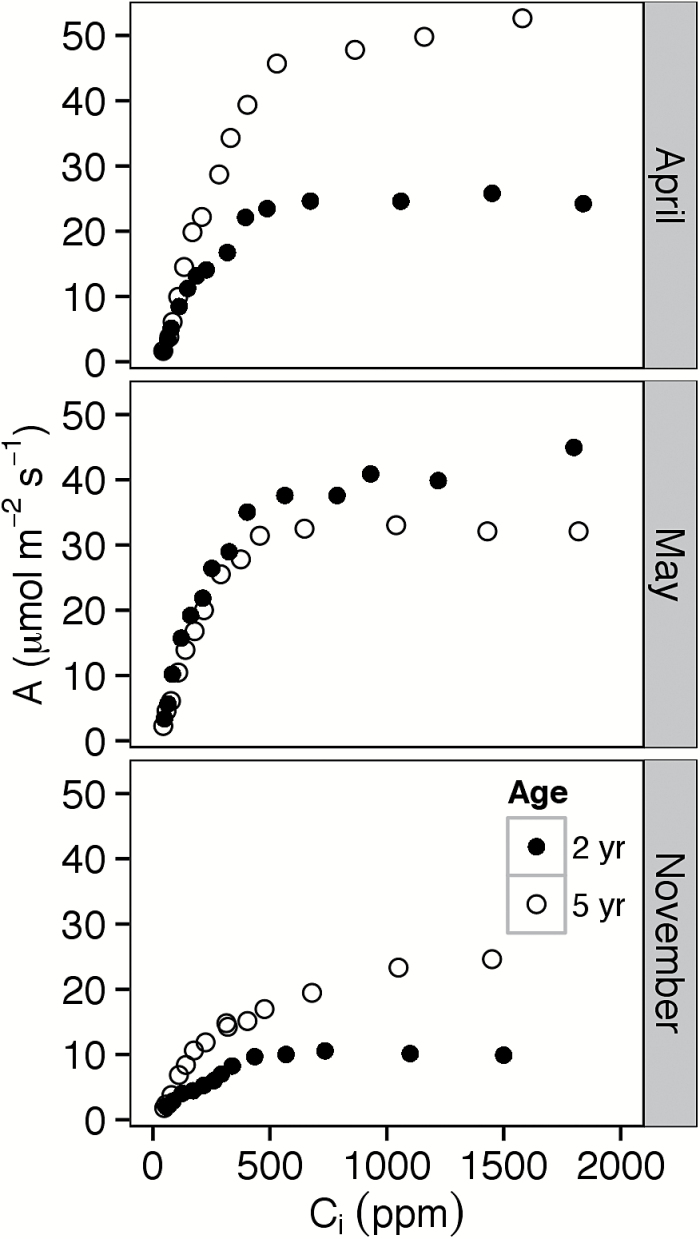
Photosynthetic rate at approximately ambient irradiance (*A*) as a function of intercellular carbon dioxide concentration (*C*_i_) in representative leaves of 2- and 5-year-old intermediate wheatgrass plants on 6 April (A; leaf temperature 13 ºC), 15 May (B; leaf temperature 18 ºC), and 20 November (C; leaf temperature 5 ºC). Data were taken from a 2015 study at the Kellogg Biological Station (Hickory Corners, MI).

**Fig. 3.**
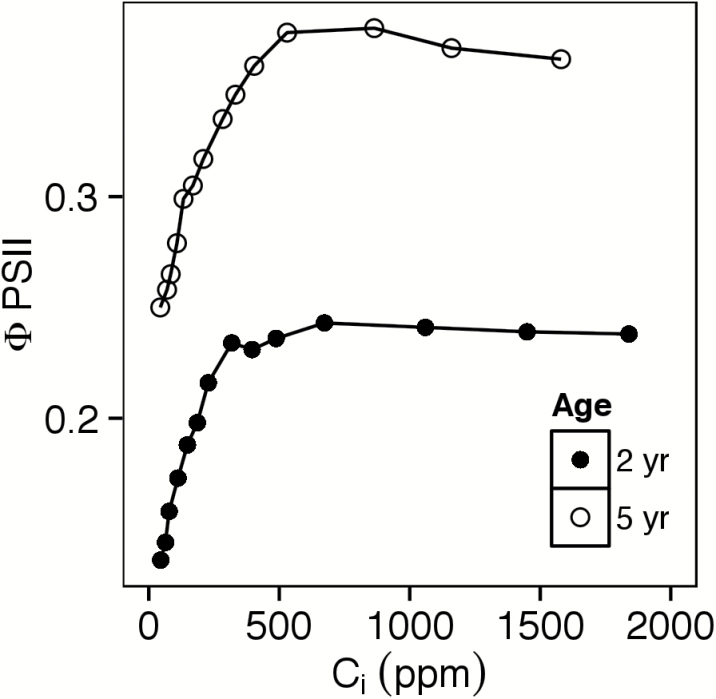
Quantum yield of PSII (ΦPSII) as a function of intercellular carbon dioxide concentration (*C*_i_) in representative leaves of 2- and 5-year-old intermediate wheatgrass plants on 6 April, with leaf temperature 12 ºC. Data were taken from a 2015 study at the Kellogg Biological Station (Hickory Corners, MI).

Representative *A*/*C*_i_ curves are shown in Fig. 2A (early April), Fig. 2B (May), and Fig. 2C (November). In ~82% of cases, TPU limitation was indicated by a decline in either *A* or ΦPSII at high *C*_i_, while in 18% of cases it was not (examples of both shown in Fig. 3). Across all three cold-stressed dates, the 5-year-old plants under cold stress exhibited smaller decreases in *A*, coupled with larger increases in temperature-adjusted TPU than 2-year-old plants (Table 2). In contrast to our expectation, under cold stress conditions temperature-adjusted *V*_Cmax_ tended to decrease rather than increase. Since these values are already temperature-corrected, this is equivalent to saying that at low temperatures, observed rates of RuBP carboxylation tended to be even lower than the Arrhenius model for temperature dependence would suggest. While the interaction term in the model for *V*_Cmax_ was not significant, a planned contrast indicated that temperature-adjusted *V*_Cmax_ decreased less in the older plants under cold stress than in the younger ones (*t*=3.76, *P*=0.0094, df=7). As described above, reported *V*_Cmax_, *J*_max_, and TPU in Table 2 are all extrapolated to 25 ºC, and are in most cases substantially different from the rates actually inferred *in vivo*: this was done to separate effects of temperature on kinetic properties of the relevant enzymes from effects of cold stress on biochemical allocation to various components of the system.

**Table 2.** Means (±SE), Student’s *t* for planned contrast, and *F*-values for photosynthetic rate (*A*), inferred maximal electron transport rate (J_max_), maximal RuBP carboxylation rate (V_Cmax_), and maximal triose phosphate utilization capacity (TPU) in 2-year-old and 5-year-old wheatgrass (Thinopyrum intermedium) at multiple sampling dates in a 2015 study at Kellogg Biological Station (Hickory Corners, MI) Values given for *V*_Cmax_, *J*_max_, and TPU are standardized to 25 ºC, while values for *A* are not. At the first three dates, second year plants were not measured.

**Date**	**Cohort**	***A* (μmol m** ^**−2**^ **s** ^**−1**^)	***J***_**max**_	***V***_**Cmax**_	**TPU**	**Temperature** (º**C**)
18 March	Wheatgrass (5-year-old)	8.89±0.9	208.8±42.9	36.5±8.5	33.5±3.2	5.2
24 March	Wheatgrass (5-year-old)	8.12±0.9	234.6±39.2	38.6±1.8	30.5±5.1	7.4
4 April	Wheatgrass (5-year-old)	5.09±0.7	135.2±30.9	35.6±9.1	28.7±3.3	2.8
8 April	Wheatgrass (2-year-old)	17.6±1.6	224.4±20.5	112.0±13.7	26.2±3.1	12.8
8 April	Wheatgrass (5-year-old)	25.5±1.3	356.8±27.7	79.7±14.3	41.9±3.5	12.8
25 April	Wheatgrass (2-year-old)	9.9±1.7	193.7±28.0	56.0±14.0	22.2±3.5	6.8
25 April	Wheatgrass (5-year-old	10.2±1.9	199.1±30.6	101.3±13.5	30.5±1.3	6.8
20 May	Wheatgrass (2-year-old)	30.8±3.4	198.4±30.4	118.4±17.1	20.7±2.7	19.9
20 May	Wheatgrass (5-year-old)	24.4±1.6	235.2±30.8	108.6±14.6	19.2±2.8	19.9
10 July	Wheatgrass (2-year-old)	28.3±3.3	142.0±18.0	93.0±8.1	14.0±1.2	28.6
10 July	Wheatgrass (5-year-old)	25.2±2.8	174.0±16.0	92.3±7.3	14.9±1.0	28.6
20 November	Wheatgrass (2-year-old)	11.4±1.6	197.1±13.2	65.6±10.3	27.1±2.0	7.5
20 November	Wheatgrass (5-year-old)	19.4±2.4	253.3±22.6	109.9±15.3	37.8±2.5	7.5
**Planned contrast**						
Student’s *t*-value		6.52****	1.80	5.34**	2.59*	
**Source of variation**						
Stress		170.39****	13.19***	3.39^†^	28.20****	
Age		0.11	6.61*	1.14	9.37**	
Age×stress		15.28***	1.97	1.26	6.60*	
Date		18.55****	7.02***	0.68	23.46****	

†, *, **, ‘***’ and ‘****’ indicate significance at α= 0.075, 0.05, 0.01, 0.001 and 0.0001, respectively.

Significance of the cold stress×age interaction may be assessed either by the *F*-value or by the planned contrast.

Under cold stress, 5-year-old plants had 66% higher *A* (*t*=5.03, *P*=0.0024, df=6) and 38% higher *V*_Cmax_ (*t*=5.31, *P*=0.0093, df*=*7). Based purely on the directly measurable values, 5-year-old plants had 37% higher TPU than 2-year-old plants (*t*=4.22, *P*=0.0007, df*=*16): when missing data were imputed as their lower bound, the older cohort had 42% higher TPU (*t*=5.14, *P*=0.0021, df=6) than the younger plants. Electron transport capacity (*J*_max_) was also 30% higher in older plants under cold stress. In contrast, under warm and non-stressed conditions in May–July, photosynthetic parameters did not differ between the cohorts. Surprisingly, in spite of the cold sensitivity of sugar phosphate synthesis, TPU limitation was most commonly seen in the warm temperature measurements. It is possible that this species undergoes intense cold acclimation in spring and autumn involving large increases in triose phosphate synthesis capacity.

### Mesophyll conductance

Estimates of *V*_Cmax_ can be sensitive to the assumed or estimated values for *g*_m:_ overstating *g*_m_ leads to underestimating the true value of *V*_Cmax_, and vice versa. This is particularly the case because the method used to estimate *g*_m_ assumes that all net electron transport is used to power RuBP regeneration, which may not be correct. The surprisingly low apparent *V*_Cmax_ in early spring, in spite of high Rubisco content, could partially reflect changes in *g*_m_ if alternative electron sinks become more important. Under the constraints of this field study, we could not estimate the fraction of electron transport allotted to non-photosynthetic electron sinks: however, we did calculate *g*_m_ for each cohort in April and May and compare these results with those based on the temperature response function. Older and younger plants had similar overall *g*_m_ values and responses to temperature (Table 3). Compared with predicted mesophyll conductance from published temperature response functions, observed *g*_m_ was ~95% higher in April 2015 and 60% lower in March 2016. While differences in *g*_m_ may contribute partially to explaining the apparent low *V*_Cmax_ at low temperature, the high values in April (coupled with surprisingly low *V*_Cmax_) suggest that it is not the only factor involved.

**Table 3.** Means (±SE) and F-values for stomatal limitation (L_S_), mesophyll conductance (g_m_), and intrinsic water-use efficiency (IWUE) in two cohorts of intermediate wheatgrass (Thinopyrum intermedium) at multiple sampling dates in a 2015–2016 study at Kellogg Biological Station (Hickory Corners, MI). Symbols ‘*” and ‘**’ represent significance at α = 0.05 and 0.01 respectively

**Date**	**Cohort**	***L***_**S**_ (%)	***g***_m_ (µmol m^−2^ s^−1^ Pa^−1^)	**IWUE** (mmol mol^−1^)
April 8	Wheatgrass (2-year-old)	27.5±4.3	5.78±1.48	100±24
April 8	Wheatgrass (5-year-old)	21.0±4.0	4.83±0.86	50±3*
10 July	Wheatgrass (2-year-old)	21.6±4.3	10.30±1.69	59±9
10 July	Wheatgrass (5-year-old)	21.0±3.1	9.87±2.35	85±20*
20 November	Wheatgrass (2-year-old)	19.1±3.2	1.33±0.32	49±3
20 November	Wheatgrass (5-year-old)	12.3±4.5	1.88±0.74	45±7
17 March	Wheatgrass (3-year-old)	–	0.50+0.15	170±21
17 March	Wheatgrass (6-year-old)	–	0.66+0.15	114±17*
**Source of variation**				
Stress		2.66	56.45***	
Age		0.5	0.03	
Age×stress		5.46*	0.06	
Date		10.88**	8.05**	

*, **, and *** indicate significance at α=0.05, 0.01, and 0.001 respectively.

### Temperature response of photosynthesis in early spring (Experiment 2)

**Fig. 4.**
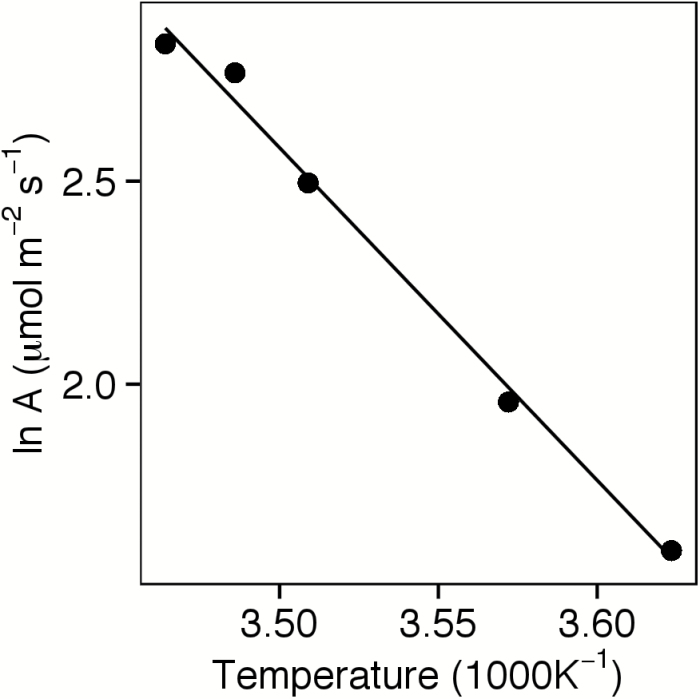
Log-transformed photosynthetic rate at approximately ambient carbon dioxide, light, and humidity, as a function of reciprocal-transformed absolute temperature in newly emerged leaves of 5-year-old intermediate wheatgrass plants. Observed photosynthetic rates are each based on the average of six plants. Data were taken from a 2015 study at the Kellogg Biological Station (Hickory Corners, MI).

Over the period 24 March–25 April in 2015, the photosynthetic rate in newly emerged leaves of fifth-year plants was predictable as an Arrhenius function of leaf temperature (*r* ^2^=0.99, *n*=5; Fig. 4) with an approximate *Q*_10_ of 2.66 over the 0–15 ºC range. Surprisingly, significant photosynthetic activity (3.3–6.9 μmol m^−2^ s^−1^) was still observed at temperatures as low as 2.8 ºC (Table 3). Overwintered leaves from the previous autumn, sampled on 18 and 24 March 2015 had photosynthetic rates (6.5–11.5 μmol m^−2^ s^−1^) ~40% higher than those predicted from the Arrhenius curve for newly emerged leaves, suggesting that these leaves lose little photosynthetic capacity over the winter and strongly acclimate to cold. Leaves sampled on 20 November 2015 also had higher rates than expected based on the Arrhenius curve for spring-emerged leaves.

### Rubisco and SPS under cold and warm conditions (Experiment 2)

**Fig. 5.**
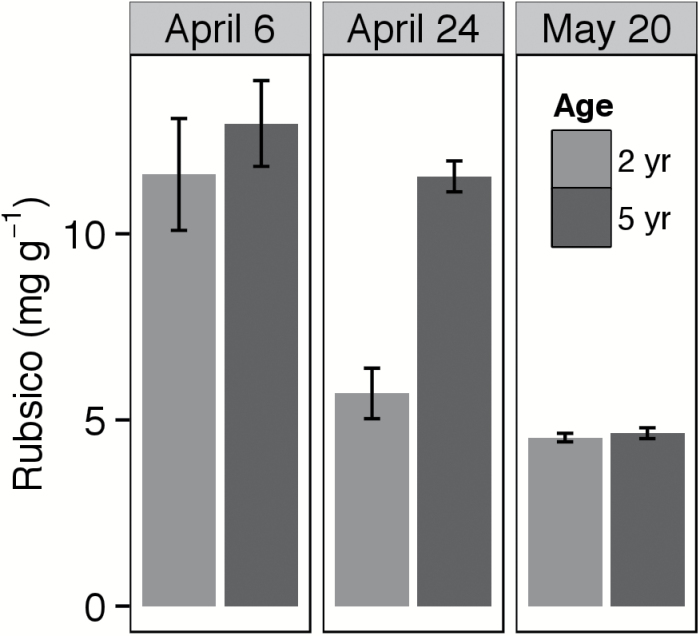
Extractable concentration of Rubisco at three sampling dates in early and mid-spring, in 2- and 5-year-old intermediate wheatgrass plants. Data were taken from a 2015 study at the Kellogg Biological Station (Hickory Corners, MI).

**Fig. 6.**
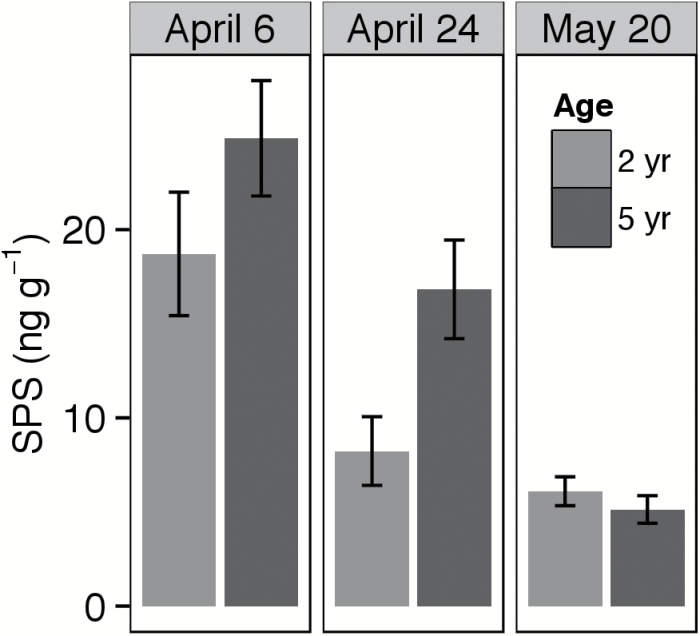
Extractable concentration of sucrose-phosphate synthase (SPS) at three sampling dates in early and mid-spring, in 2- and 5-year-old intermediate wheatgrass plants. Data were taken from a 2015 study at the Kellogg Biological Station (Hickory Corners, MI).

Age and cold stress had a strong interactive effect on levels of Rubisco (*F*=4.74, *P*=0.028) and SPS (*F*=6.06, *P*=0.015). As hypothesized, 5-year-old plants exhibited greater increases in both Rubisco and SPS under cold stress. In April, fifth-year plants had 52% more Rubisco (*t*=4.05, *P*=0.01, df=5; Fig. 5) and 77% more SPS (*t*=3.99, *p*=0.007, df*=*6; Fig. 6), consistent with the lower *V*_Cmax_ and TPU in younger plants at this time point. Rubisco levels were higher in early April than in late April, surprisingly given the lower temperature-corrected *V*_Cmax_ observed in early April. In the older cohort, SPS and Rubisco concentrations were 17–33ng g^−1^ and 10–16mg g^−1^, respectively (on a fresh weight basis) in early April and declined as temperatures increased.

### Photosynthetic response to extreme cold stress (Experiment 2)

**Fig. 7.**
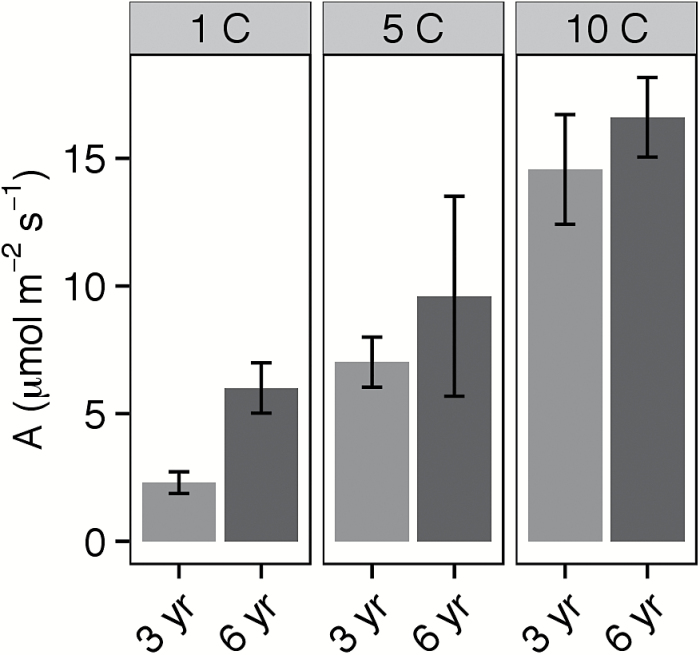
Photosynthetic rate at approximately ambient irradiance (*A*) in 3- and 6-year-old intermediate wheatgrass plants at three leaf temperatures (~1, 5, and 10 ºC) measured in the field. Data were taken from a spring 2016 study at the Kellogg Biological Station (Hickory Corners, MI).

The 6-year-old and 3-year-old cohorts showed markedly different photosynthetic performance under cold-stressed conditions in early spring 2016. Photosynthetic rate was higher in older plants (*F*=6.85, *P*<0.05), and declined at lower temperature (*F*=39.69, *P*<0.0001), but there was no interaction of age and temperature within this range. Effects of low temperature on PSII efficiency also strongly differed between cohorts. At 1.2 ºC, the 6-year-old plants maintained over twice the photosynthetic rate (*t*=4.73, *P*=0.002, df=7; Fig. 7) of the 3-year-old plants, and similar differences were seen with respect to PSII efficiency (*t=*4.38, *P*=0.0047, df=6; Table 4). Consistent with our observations in April 2015, older wheatgrass plants showed significant photosynthesis even at temperatures approaching freezing at the leaf surface.

**Table 4.** Means (±SE) and F-values for the ratio of variable to maximal fluorescence in the light (F_v_^'^/F_m_^'^), PSII efficiency (ΦPSII), intrinsic water-use efficiency (IWUE), and imputed triose phosphate utilization (TPU; estimated as the carbon-saturated photosynthetic rate at 2000 μmol mol^−1^) in 3-year-old and 6-year-old intermediate wheatgrass (Thinopyrum intermedium) at three dates (differing in leaf temperature) in a spring 2016 study at Kellogg Biological Station (Hickory Corners, MI)

Date	Cohort	*F*_v_^'^/*F*_m_^'^	ΦPSII	IWUE (mmol mol^−1^)	TPU (imputed; μmol m^−2^ s^−1^)	Temperature (ºC)
14 March	Wheatgrass (6-year-old)	0.368±0.021	0.050±0.007	42±9	14.7±1.9	1.2
14 March	Wheatgrass (3-year-old)	0.376±0.019	0.024±0.001*	12±4	11.0±1.3	1.2
3 April	Wheatgrass (6-year-old)	0.399±0.023	0.096±0.010	109±29	–	5.1
3 April	Wheatgrass (3-year-old)	0.474±0.033	0.078±0.008	88±22	–	5.1
17 March	Wheatgrass (6-year-old)	0.452±0.011	0.138±0.016	170±21	–	9.7
17 March	Wheatgrass (3-year-old)	0.455±0.054	0.139±0.006	110±43	–	9.7
**Source of variation**						
Temperature		6.65**	63.30****	23.3****	–	
Age		0.65	12.10**	8.27**	2.53	
Age×temperature		0.56	10.91***	0.40	–	

*, **, ***, and **** indicate significance at α=0.075, 0.05, 0.01, and 0.001, respectively.

### Water relations (Experiments 1 and 2)

Water relations showed no consistent trend. Water-use efficiency was higher in younger plants in April 2013, but did not differ in May or July, and was actually 36% higher in the older plants under cold stress in 2015 (Table 3) and 82% higher in 2016 (Table 4). Stomatal limitation (*L*_S_) declined with age in 2013 but not in 2015. No clear trends were seen for Ψ_L_: second-year plants had lower pre-dawn Ψ_L_ than first-year plants, but third-year and first-year plants did not differ. Pre-dawn water potential ranged between –0.16MPa and –0.22MPa. Mid-day water potential and diurnal change in water potential (Ψ_L,MD_ *–*Ψ_L,PD_) did not vary among cohorts (Table 1).

## Discussion

In general, our second and third hypotheses (regarding improved cold tolerance in older plants) were supported. Under cold stress, older cohorts achieve superior performance to younger ones, exhibiting higher *V*_Cmax_, *A*, and TPU than younger plants under cold stress (supporting the second hypothesis). Likewise, they had greater levels of Rubisco and SPS than younger plants under cold stress but not at mid-season (supporting the third hypothesis). As expected, [Rubisco], [SPS], and TPU (corrected for temperature) declined over the 2015 season, consistent with much previous work that suggests that activity or amounts of these parameters increase as part of acclimation to cold (Bascunan-Godoy *et al.*, 2006), including in cold-season grasses such as rye (Hurry *et al.*, 1994) and wheat (Savitch *et al.*, 1997; Yamasaki *et al.*, 2002). Surprisingly, temperature-corrected *V*_Cmax_ showed an increase over the course of the season, in contrast to the Rubisco data (alternatively, at low temperature, RuBP carboxylation was lower than the Arrhenius model would predict). While this is difficult to explain, it may reflect serious limitation to the activation state of Rubisco under extreme cold that is not accounted for by the standard model. Some evidence from the warm-weather species sweet potato (*Ipomoea batatas*) suggests that Rubisco may become deactivated at suboptimal temperature to compensate for phosphate limitation (Cen and Sage, 2005). As noted above, discrepancies in *g*_m_ or alternative electron sinks could also explain part of the difference.

Evidence regarding our first hypothesis was more equivocal. Under optimal growing conditions at mid-season, older wheatgrass plants experienced decreased photosynthetic rates relative to younger plants in the first experiment, but exhibited similar photosynthetic capacity to younger plants in the second. In contrast to plants such as black cherry (Fredricksen *et al.*, 1996), the age-related photosynthetic decline in our first experiment did not reflect more conservative water use (evident from the lack of consistent differences in WUE or Ψ_L_ at mid-season, coupled with lower *L*_S_ in older plants). Rather, the older wheatgrass plants in this experiment had lower biochemical capacities for carbon assimilation. Investing in cold tolerance has generally negative impacts on growth (due both to resource trade-offs and to biochemical pathways involved in stress tolerance which depress growth), and it is plausible that this helps explain part of the observed yield decline with age (Canode, 1965). Our results are consistent with the idea that as some herbaceous perennials age, they gain increased resistance to abiotic stresses, possibly at the cost of lower photosynthetic capacity under optimal conditions, or lower growth rate (Bond, 2000; Gusta and Wisniewski, 2013). This study builds on work that has accumulated over the past few decades indicating that many woody perennials show declines in photosynthetic capacity with whole-plant age, and often show compensating increases in cold tolerance (MacNamara and Pellet, 2000; Lim *et al.*, 2014; Miguez *et al.*, 2014). It also parallels recent work on the C_4_ perennial grass *Miscanthus*×*giganteus* which suggests that older plants prioritize remobilizing nutrients in preparation for winter freezing tolerance, at the expense of lower photosynthetic capacity in the autumn (Boersma *et al.*, 2015).

Beyond the central finding of this study, indicating trade-offs between cold tolerance and growth with age, some additional observations deserve comment. One interesting note was that we found significant photosynthesis (15–19% of peak) at near-freezing temperatures (leaf temperature of 2.8 ºC and 1.2 ºC, corresponding to air temperatures of ~0 ºC). This compares with 30% of peak photosynthesis at 0 ºC in the Antarctic species *Colobanthus quitensis* (Xiong *et al.*, 1999, 2000). Unlike this extreme stress tolerator, however, *Thinopyrum* has high photosynthetic capacity in general and also performs well at moderate temperatures. Relatively few studies of temperature response in plants have considered extremely low temperatures: exceptions include *Colobanthus* and the alpine herbaceous evergreen *Saxifraga paniculata* (Hacker and Neuner, 2006). Eddy covariance data suggest that some cold-season grasses may have minimum temperature requirements for photosynthesis as low as –4 ºC (Skinner, 2007). A second observation was that wheatgrass leaves entering or emerging from winter maintained higher photosynthetic rates under cold conditions than newly emerged spring leaves. In this species, leaves that expand in autumn and last through the winter seem to lose very little if any photosynthetic capacity, and to acclimate strongly to the cold. Finally, we found clear evidence that TPU capacity in this species could limit photosynthesis, especially in younger plants. TPU limitation of photosynthesis may be an under-rated limitation under cold conditions, particularly as global [CO_2_] continues to increase.

Intermediate wheatgrass, as a stress-tolerant, productive, and nutritious relative of the world’s third most widely grown crop, has existing and growing economic importance. More generally, herbaceous perennials are a very widespread mode of plant life history in both natural grasslands and pastures. Understanding how photosynthetic rates and stress tolerance change with age may have implications for modeling long-term productivity and resource uptake in these systems.

## Abbreviations:

A: photosynthetic assimilation of carbon dioxide under outside conditions
*C*_i_: intercellular [CO_2_]
*C*_C_: chloroplastic [CO_2_]
*F*_v_'*/F*_m_*'*: ratio of variable to maximal fluorescence in the light
*g*_m_: mesophyll conductance
*g*_s_: stomatal conductance
*J*_max_: maximal electron transport rate
*K*_C_: Michaelis constant for RuBP carboxylation
*K*_O_: Michaelis constant for RuBP oxygenation
*L*_S_: degree of stomatal limitation
SPS: sucrose-phosphate synthase
TPU: triose phosphate utilization
*V*_Cmax_: maximal RuBP carboxylation rate
Γ*: photocompensation point;
ΦPSII: photosystem II efficiency
Ψ_L_: leaf water potential.
